# A Novel Hybrid Approach Based on Deep CNN Features to Detect Knee Osteoarthritis

**DOI:** 10.3390/s21186189

**Published:** 2021-09-15

**Authors:** Rabbia Mahum, Saeed Ur Rehman, Talha Meraj, Hafiz Tayyab Rauf, Aun Irtaza, Ahmed M. El-Sherbeeny, Mohammed A. El-Meligy

**Affiliations:** 1 Department of Computer Science, University of Engineering and Technology Taxila, Punjab 47050, Pakistan; rabbia.mahum@uettaxila.edu.pk (R.M.); aun.irtaza@uettaxila.edu.pk (A.I.); 2 Department of Computer Science, COMSATS University Islamabad-Wah Campus, Wah Cantt 47040, Pakistan; srehman@ciitwah.edu.pk (S.U.R.); talha_cui@ciitwah.edu.pk (T.M.); 3 Department of Computer Science, Faculty of Engineering & Informatics, University of Bradford, Bradford BD7 1DP, UK; 4 Industrial Engineering Department, College of Engineering, King Saud University, P.O. Box 800, Riyadh 11421, Saudi Arabia; aelsherbeeny@ksu.edu.sa (A.M.E.-S.); melmeligy@ksu.edu.sa (M.A.E.-M.)

**Keywords:** feature extraction, sensor based HR imagery, knee osteoarthritis, Convolution Neural Networks, knee osteoarthritis detection

## Abstract

In the recent era, various diseases have severely affected the lifestyle of individuals, especially adults. Among these, bone diseases, including Knee Osteoarthritis (KOA), have a great impact on quality of life. KOA is a knee joint problem mainly produced due to decreased Articular Cartilage between femur and tibia bones, producing severe joint pain, effusion, joint movement constraints and gait anomalies. To address these issues, this study presents a novel KOA detection at early stages using deep learning-based feature extraction and classification. Firstly, the input X-ray images are preprocessed, and then the Region of Interest (ROI) is extracted through segmentation. Secondly, features are extracted from preprocessed X-ray images containing knee joint space width using hybrid feature descriptors such as Convolutional Neural Network (CNN) through Local Binary Patterns (LBP) and CNN using Histogram of oriented gradient (HOG). Low-level features are computed by HOG, while texture features are computed employing the LBP descriptor. Lastly, multi-class classifiers, that is, Support Vector Machine (SVM), Random Forest (RF), and K-Nearest Neighbour (KNN), are used for the classification of KOA according to the Kellgren–Lawrence (KL) system. The Kellgren–Lawrence system consists of Grade I, Grade II, Grade III, and Grade IV. Experimental evaluation is performed on various combinations of the proposed framework. The experimental results show that the HOG features descriptor provides approximately 97% accuracy for the early detection and classification of KOA for all four grades of KL.

## 1. Introduction

Osteoarthritis (OA) is a severe disease in joints, especially in the knees, due to loss of cartilage. It appears with age, and it is present mostly in the elderly population. Overweight is also among the various causes of the prevalence of OA [1,2]. The Knee joint consists of two major bones, the femur and the tibia. Between these bones, a thick material called cartilage is present. This cartilage helps with the flexible and frictionless movement of the knee. Cartilage volume may decrease due to aging or accidental loss [3]. Due to decreased cartilage volume, tibiofemoral bones produce friction during movement, leading to knee osteoarthritis (KOA). Articular cartilage is composed of a chondrocyte that helps the underlying bone by load distribution, and it works for a lifetime [4].

Kellgren–Lawrence (KL) is a grading system that describes the various stages of OA. This system is based on the radiographic classification of KOA. It is found to be the most authoritative system of classification. It consists of Grade I, Grade II, Grade III, and Grade IV [5]. Early symptoms that indicate KOA in patients are knee pain, swelling, surface roughness, gait abnormalities, morning pain, and so forth. From these factors, doctors detect the presence of the disease. Although KOA is detected below the age of forty years, the average age of the patients has been reported to be above forty-five years [6]. According to a recent study, 80% of people over the age of 65 have radiographic KOA in the USA [5]. It is expected that the ratio will increase in the future. Another study has stated that KOA affects more than 21 million people in the USA [7]. In Indonesia, 65% of total arthritis cases are knee osteoarthritis [8]. In Asia, it is also increasing day by day. According to a recent study conducted in Pakistan, 28% of the urban and 25% of the rural population is affected by knee osteoarthritis [9]. Clinically, along with medication, KOA is cured by exercise, weight loss, walking aids, heat and ice treatment, and physiotherapy as non-invasive methods and acupuncture, intra-articular injection, and surgical procedures as invasive methods of treatment [10].

Image processing is a computer-aided technique that is used for KOA detection. Various modalities, such as radiography, MRI, gait analysis, bioelectric impedance signals, and so forth, are used for the detection of KOA [11,12]. X-rays/radiographic images help to detect knee osteophytes and joint width space narrowing, while MRI is helpful for cartilage thickness detection, surface area, and roughness. In contrast, bioelectric impedance signals are a powerful tool for the detection of KOA. As it is a non-invasive technique, it is low cost and easy to operate. It involves the recording of electrical signals around the knee. Later, these signals are used for the analysis and detection of KOA [13]. Radiography is a simple and cheap procedure for the detection of KOA. Through it, we can see the joint space width easily. It is used almost everywhere in the world as it is a cheap modality. However, it has a limitation in that we cannot see the details of the image, and it does not provide any information for the early detection of KOA [13]. The MRI technique is more advanced than radiography in the detection of the morphological features of the knee. It provides an in-depth image of the structure and formation of the knee. We can obtain useful information using image processing techniques on MR images. However, it is costlier than Radiography and can be more useful [13,14]. The image processing techniques, such as segmentation, thresholding, masking, edge detection, contrast enhancement, and so forth, are applied for obtaining the required data from the images.

Various machine learning and deep learning techniques have been used for the detection of KOA using images of radiography [2,3,15,16,17,18]. Deep learning algorithms are usefulness in various domains such as for mission-critical applications [19,20], semantic segmentation [21], medical, that is, real-time cardiovascular Magnetic Resonance [22], and ecosystems change analysis [23]. Deep learning algorithms perform very well in the medical field. However, deep learning techniques did not perform well for KOA classification using radiographic images. Although these algorithms performed well for binary classification among OA and non-OA images with an accuracy of 92% but for multi-classification the accuracy was 66.7% [15].

Therefore, this study proposes a novel technique for KOA detection according to the KL grading system. The technique uses a hybrid approach for feature extraction, and classification is performed with three different multi-class algorithms—SVM, KNN, and Random Forest. The result for the KNN classifier is better than that of the others.

The remaining sections of papers are organized as follows: Section 1 refers to the Introduction, Section 2 refers to the Literature Review, Section 3 refers to the Proposed Methodology, and Section 4 and Section 5 refer to the Experimental evaluation and the Conclusion.

## 2. Literature Review

OA is a common joint disorder. It appears with aging and also due to wear and tear on joints. Overweight persons have an increased risk of OA in different joints [1]. Osteoarthritis causes the degradation of articular cartilage, which is a flexible coating between the knee bones. OA causes mechanical abnormalities of the knee and hips. In this method, gait analysis is performed to predict joint deterioration [24]. Joint mechanics and function are based on the efficient working of menisci. These menisci enable load balancing at tibia-femoral bones. It also facilitates articular cartilage by reducing the load on it. The lubrication and distribution of synovial fluids are also regulated and affected by menisci [25]. There are two types of material from which knee bone is made; one type of material is known as Cancellous or Trabecular (Spongy) bone, and the other is known as Cortical (compact) bone [26]. The bone has different shapes; some bones are long, some are short, some are flat, and other bone shapes are irregular [27]. They have presented a Layered graph approach for optimal segmentation. It can be applied on single and multiple interacting surfaces [28].

Mosaicplasty is a self-cartilage transplantation method. In the case of knee cartilage damage, it is one of the remedies. It requires 3D image precision [29]. Osteoarthritis and rheumatoid arthritis are other widespread diseases that are inclined to cause effusion. Even situations, such as gout or the formation of tumors and cysts, can trigger fluid keeping in and around the knee. A fully automated segmentation technique is used. This technique uses MR images and is applied for the detection of osteoarthritis of the knee [14,30]. Image processing techniques, such as histogram quantization, threshold, region of interest processing, edge detection, and so forth, are used to detect the breakdown of the cartilage [11,12]. Image processing techniques in medical diagnosis are presented. Edge detection and contrast enhancement are shown based on the threshold. This threshold directly affects the results. These experiments are performed on the Linux platform using the C language. The proposed algorithm helps in the case of noisy and blurred images [31]. A fully automated method for the segmentation of cartilage and bone is performed on MRI images. Cartilage volume, thickness, and surface area are detected based on knee segmentation. These parameters are then used for the progression of KOA [32].

Fully automated bone segmentation using a Graph cut algorithm has been used. The images used in this technique are taken from the OAI publicly available database. Here, MR images are used and classified to detect the bones, background, and to detect the fats present in the MR images. In this study, a two-phase approach has been proposed. In the first phase, areas of bones (femur and tibia) are identified. The output of the first phase is given as input to the second phase, where bone segmentation is performed, and other structures, such as fat and muscle, are separated. The accuracy of detection in the first phase is 0.99, whereas, in the second phase, accuracy is 0.95 mean DSI [33]. In addition, the cartilage composition is assessed by MR imaging. In this work, they have developed a direct segmentation technique (DST) to detect knee osteoarthritis. The imaging data have been taken from the OAI database [34]. Used X-ray images for automatic detection of KOA. They have detected different forms such as standard, doubtful, minimal, and moderate KOA. The dataset consists of 350 X-ray images. The KL classification is done manually. In this method, image features are first extracted. This process is carried out on transforms. For better results and feature extraction, transformation is also used. Experimental results showed that average and minimal grade KL OA was easily differentiated from normal OA with an accuracy of 91.5% and 80.4%, respectively, while doubtful OA was detected with an accuracy of 57% [5]. In their work, they have used fully automated segmentation techniques that have used three-label bone segmentation. They have also applied a convex optimization technique for the segmentation of knee cartilage. The proposed method provides a more significant result than manual segmentation [35]. A new graph cut technique is used for the detection of KOA, in which MR images are used for the segmentation [33].

A semiautomatic technique is used on knee MRI images to obtain a segment of cartilage. The cartilage segment is separated from femur and tibia bones [11]. In this study, a computer-aided image analysis method is used to detect the early development of KOA. This method detects the texture and structural changes in an image, such as bone-in-knee and hip KOA. Radiographic images or X- rays are used in this method. First, X-rays are taken and then digitized. The joint detection is automatically performed, and common areas are then separated from the image. Numerical image features or content descriptors are then extracted. In the end, images are classified based on the feature values. For moderate KOA (KL-3), the experimental results have shown an accuracy of 72%. While for mild KOA (KL-2), the accuracy was 62%. The critical aspect of the research is that the part of the tibia just beneath the joint is very informative for the early detection of KOA. This part has produced substantial and higher signals. Other areas of the tibia and femur away from the joint did not produce any signal and hence are less helpful in the early detection of KOA [36]. In [6], for early KOA detection, an automated technique is proposed using X-ray images. Firstly, images are preprocessed using the circular Fourier filter. Then, multi-linear regression is applied to minimize the variations among healthy and OA parts. Then for feature extraction, an independent component analysis is used, and at the end, Naïve Bayes and random forest classifiers are used to detect KOA. The algorithm gave 82.98% accuracy and 80.65% specificity. Knee OA is detected by using the knee joint space parameter [2]. The region of interest is separated through template matching using the HOG feature vector, then the knee joint space is calculated and compared with the reference knee joint space width. Detail of related works on the detection of Knee Osteoarthritis is given in Table 1.

However, the method only detects the KOA, showing an accuracy of 97%. In [46], a region-based technique was used to detect the KOA. Histograms of gradient elements were calculated using a multi-class support vector machine (SVM), and results were categorized based on Kellgren and Lawrence’s (KL) grading system. Accuracies of <98% for Grade-0, 93% for Grade-I, <87% for Grade-II, and 100% for Grade-3 and Grade-4 were attained. In [47], Hu’s moments were used to extract information by understanding the geometric transformation of the cartilage from distorted images. Total seven invariants were calculated. At last, classification was performed using K nearest neighbor (KNN) and the decision tree classifier. KNN performs better than the decision tree and gives an accuracy of 98.5%, approximately. Nevertheless, the proposed system used 2000 X-ray images for training and testing.

From the literature discussed above, it is observed that various studies proposed techniques that worked on knee X-ray images for the detection of KOA or their own created datasets. To attain good results, many authors used morphological processing on images and feature extraction and classification algorithms such as HOG, Hu’s, SVM, and KNN. The contributions of this study are below:

- To propose a novel robust algorithm that can carry out early detection of KOA according to the KL grading. The proposed algorithm uses X-ray images for training and testing the results. The hybrid features descriptors extract features, that is, CNN with HOG and CNN with LBP. Three multi-classifiers are used to classify disease according to the KL grading system (I, II, III, IV), such as KNN, RF, and SVM;
- Cross-validation has been used, using 420 images to evaluate the performance of the proposed technique, and results show 97% accuracy for overall detection and classification;
- A five-fold validation is used, such as (50,50), (25,75), (30,70), (40,60), (20,80); here, an individual set represents the train and test data respectively for each Grade and the last set is for a healthy class. Our proposed technique gives an accuracy of 98% for all grade classifications;
- We analyzed the performance of individual grade detection during cross-validation, revealing the following facts for the classification: The algorithm obtained 98% accuracy for Grade I, 97% accuracy for Grade II, 98.5% accuracy for Grade III and Grade IV;
- Due to the algorithm’s robustness, it can be used for other disease detection and classification, acquiring significant results.

## 3. Proposed Framework

The first step of the proposed system is preprocessing to detect the contours of the knee and to remove noise. Then region of interest is extracted, and segmentation is carried out. In the third step, features are extracted using Deep Convolutional Neural Network (DCNN) hybridized as Convolutional Neural Network (CNN), Histogram of Oriented Gradient (HOG), and DCNN with Local Binary Patterns (LBP). Features are extracted as texture, shape, scaling, rotation, and translation. These extracted features are passed to multiclass classifier Support Vector Machine (SVM), K nearest neighbors (KNN), and Random Forest to classify the images into four grades according to the KL grading system. The detail of the proposed deep learned system is given below:

### 3.1. Pre-Processing

The aim of preprocessing images is to prepare the data for further processing in the proposed system. Format conversion is performed in this step while improving the image quality. Images are converted into TIFF format because it preserves the overall quality of the images by storing the image information without loss. During the conversion, irrelevant information is removed. In addition, a negative of the image is formed, as it enhances the visibility of the region of interest. Finally, images are required to downscale using a bilinear approach from all dimensions to improve computational complexity. It also minimizes the noise as in the bilinear approach; the output value of the pixel is the average of weights of pixels in 2 × 2 neighborhoods.

### 3.2. Region of Interest (ROI) and Segmentation

The key aspect of the algorithm is detecting the early KOA disease space width of the knee joint. This disease becomes advanced as the gap between knee joints increases with age. The region of interest (ROI) is the tibiofemoral joint. The ROI is calculated through a matching technique with the database of knee images. The database image moves on the input image pixel by pixel, and the similarity among the image’s blocks through a histogram of gradients’ features is computed. The block, having maximum similarity, is selected as the ROI. This similarity-based mechanism outperforms the traditional algorithms. Let us suppose that an input image *I* of knee is fed to the system having size *I × J*, and *D* represents the database image, having size $d_{r}$ × $d_{c}$, where $v^{d\;}$ is the vector of HOG, having a size of 1 × *h* of the database image *D.* and $s_{m,n\;}$ is the block of *d*${}_{r}$ × $d_{c\;}$ that is located at (*m,n)* in the image *I.* The HOG feature of $s_{m,n\;}$ is represented as $V_{m,n}^{s}$. Mean absolute difference (MAD) is used to compute the similarity among the database image *D* and the image block $s_{m,n.}$

(1) $$U_{m,n}=\;\frac{1}{h}\sum_{l=1}^{h}\left[V_{m,n}^{s}\;\left(l\right)-V^{d}\right].$$

**Figure 1:**
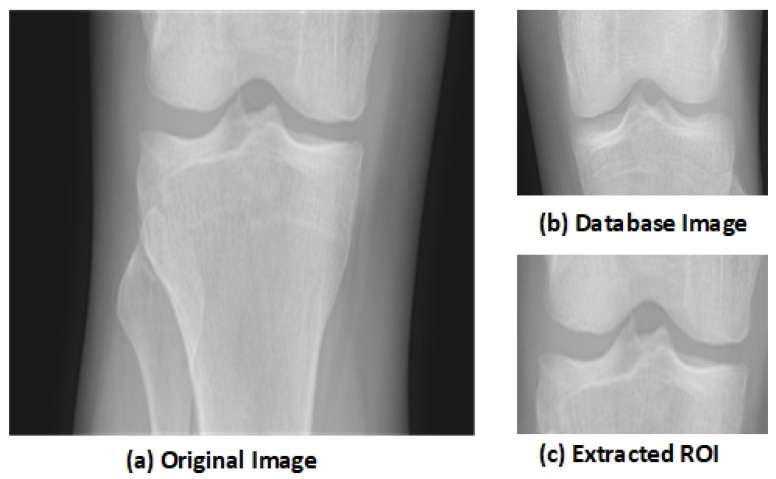
Extraction of Region of Interest (ROI) [48].

The block with the minimum MAD is selected as the ROI that contains the knee joint. The knee image that is used in the database is shown in Figure 1b. Figure 1a shows the original knee image, while Figure 1c shows the selected ROI. The selected region has essential features as it shows the joint space width (JSW).

After extracting the ROI, this cropped image is given as an input for performing the segmentation through the active contour algorithm. The image is dynamically segmented using 3 × 3 masks [49].

### 3.3. Deep Learning

Deep Learning performs nonlinear transformation hierarchy-wise. Convolutional Neural Network (CNN) has deep architecture in a feed-forward manner on which learning can be applied. Each layer in CNN can see the features and show high variance [50]. During the testing phase of the deep convolutional network, it runs in the forward direction, and all layers are distinguished. The main characteristic of deep CNN is to perform each possible match among images. There are convolutional layers that linearize manifolds while pooling layers collapse them. At the output, layer size depends upon the stride. The filter is for sharpening the image. If the kernel size is K × K and the input size is S × S, the stride is 1. The feature maps of the input are F and of the output are O, then the input size will be F@ S × S, the output size will be O @ (S−K + 1) × (S−K + 1), the kernel has the F × O × K × K coefficients that must be learned, and the cost will be F × K × K × O × (S−K + 1) × (S−K + 1). The filter size should be matched with the size of the output pattern that is to be detected. The stride between the pools is the factor on which the output size depends. For example, if independent pools have a size of K × K and the input size is S × S with F features, then the output will be F @ (S/K) × (S/K).

The output function is defined as in the below equation:

(2) F=FLoFL−1o..........F1,

where FL refers to the layer that considers the output *o* of the previous layer as an input, represented by × L -1 to calculate the output × L depending upon the weights *ω*L for every single layer as in the below equation:

(3) xL=FL(xL−1;ωL)forL=2,3,....L.

### 3.4. Feature Extraction

Features are extracted based on the shape that depends upon the knee joint space. The extracted level features include area, compactness, perimeter, lengths, that is, maximum and minimum axial length, circulatory, diameter, and convex area. Low-level features are computed through HOG, while texture features are computed using the LBP descriptor. On the other hand, high-level features are computed through ConvNet, such as scaling, rotation, and translation. In existing techniques, single or separate feature descriptors have been used that somehow fail to classify all grades of KOA due to KL having more than 95% accuracy [46]. In our proposed technique, both low and high-level features are used for the resultant image that outperforms the state-of-the-art by complete matching with the trained knee features.

### 3.5. Convolutional Neural Network as Feature Descriptor

**Figure 2:**
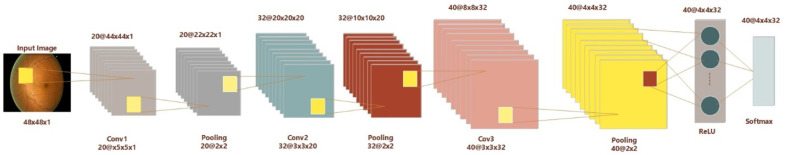
Deep CNN Architecture for the Input Image.

In our proposed system, the first layer of a 2-dimensional CNN is a convolutional layer that has a filter size of 20 while the stride size is 1. It is followed by the max-pooling layer with a size of 2 × 2 and a stride of 1. The next layer is a convolutional layer with a stride size of 1 and a filter size of 32. All the first six layers are arranged alternatively as the convolutional and max-pooling layers. The next seventh layer is the activation layer that has a Rectified Linear Unit (ReLU), while the next layer is the convolutional layer with a filter size of 40 (4 × 4 × 32). The final layer is a softmax function layer. The weights of the convolutional layers and the values of operators in max-pooling layers should be steady for valuable computations. In our datasets, the image size is 50 × 50 × 1, which converts into a size of 1 × 1 × 2 with the help of forwarding propagation of all layers [51]. Deep Neural Network layers used for the proposed technique are shown in Figure 2. CNN has convolutional layers that take the input of image *I*, filters are applied as *f* having dimensions as *m × n* with length *l* weights as *w* and bias as *b*. So, the output can be written in the form of an equation as below:

(4) $$\left(I*f\right)x,y=\sum_{s=1}^{l}\sum_{t=1}^{w}f_{st}\;\times I_{x+s-1,y+t-1}+b.$$

### 3.6. Histogram of Oriented Gradient

The images are converted into sizes from 28 × 28 to 6 × 6 concurring blocks, and each block has a size of 2 × 2 with a stride of size 4. A total of nine bins are formed. A total of 1296 low-level features are computed. Normalization can be performed for better feature extraction as pulmonary images show better intensity and shadow normalization. Intensity is considered in the blocks of a greater image size. A similar orientation is computed for the opposite directions of the image part as they are grouped in the same bin. The range of the angle remains from 0 to 180. Gradient Magnitude M of the pixel (x,y) and its direction is given in the below equation as:

(5) $$M\;\left(x,y\right)=\;\sqrt[{2}]{{I_{x}\;}^{2}+{I_{y}}^{2}}$$

(6) $$\vartheta =\;\frac{I_{y}}{I_{x}}\;,$$

where the angle ϑ varies from 0 to 2 π, while $I_{y}$ and $I_{x}$ are the gradients in a direction of *x* and *y*.

### 3.7. Local Binary Pattern

An LBP descriptor is used for texture feature extraction. It works on the principle that an individual pixel compares itself with its neighbor pixels; as a result, it encodes the local neighbors using the threshold function [52]. If the gray value of the neighbors is greater than or equal to the center pixel, the value of the center pixel is fixed as 1. Let k represent the total neighboring pixels, so LBP generates its double feature vectors such as $2^{16}$ = 65,536 feature vectors, and the number increases as the size of the neighboring pixels increases. The equation of the LBP is given below.

(7) $$LBP=\sum_{i=0}^{1}k\left(p_{i}-c_{i}\right)\times 2^{i},$$

(8) $$k\left(x\right)=\left\{\begin{array}{c} 1\\ 0 \end{array}\right.\begin{array}{c} \;\;\;\;\;\;\;\;\;\;\;\;\;if\;x\geq 0\\ \;\;\;\;\;\;\;\;\;\;\;\;\;otherwise \end{array},$$

where *p* is the neighbor pixel and c refers to the center one. Pseudo code for proposed framework is given in Algorithm 1.
**Algorithm 1** Pseudo code for proposed framework.**Input: $\mathrm{Images}=\left\{I_{1},I_{2},I_{3},\cdots \cdots \cdots \cdots I_{k}\right\}$****Output:ClassifiedImages****Begin****data(i) ←  1....k****While(data(i)!= eof)****{****Preprocessing of the Images $I_{k}$ (change format, downscaling, negative of the image)*****CNNF← 2DCNN Features Extraction******HOGF ← Histogram of Oriented Gradient Feature Extraction******LBPF ← Local Binary Pattern Feature Extraction******FV ← (CNNF HOGF LBPF)******FV1 ← (CNNF+HOGF CNNF+LBPF)******CL ← AssignClassLabels (Grades I...IV, Healthy)******Classification ← ( SVM (FV1, CL, testImages) KNN (FV1, CL, testImages) RF (FV1, CL, testmages) )******For j=1...n*****{*****if (Classification(j))*****← 1*****print Grade-I******else if(Classification(j))*****← 2*****print Grade-II******else if(Classification(j))*****← 3** ***print Grade-III******else if(Classification(j))*****← 4*****print Grade-IV******else if(Classification(j))*****← *Healthy******print KOA not detected*****}*****End***

### 3.8. Classification

**Figure 3:**
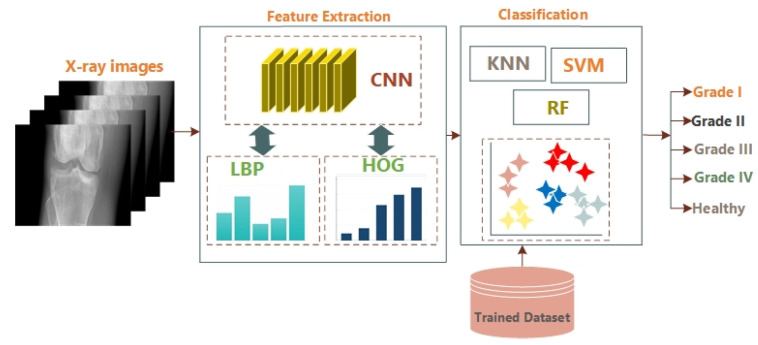
Block Diagram of the Proposed Algorithm.

For the classification, Support Vector Machine is a supervised learning algorithm trained with four classes according to the KL grading system, such as Grade-I, Grade-II, Grade-III, and Grade-IV using extracted features and the last class that belongs to a healthy knee. SVM is one of the most memory-efficient techniques. Random Forest (RF) has multiple de-correlated decision trees and remains the best for large datasets. K-Nearest Neighbour classifier (KNN) is also used and is the simplest of them. It classifies the data using a voting system and recognizes the undefined data. In KNN, if k = 1, the current object is allocated to the nearest neighbor class. The Block diagram of the proposed model is given in Figure 3.

## 4. Experimental Evaluation

### 4.1. Dataset

**Figure 4:**
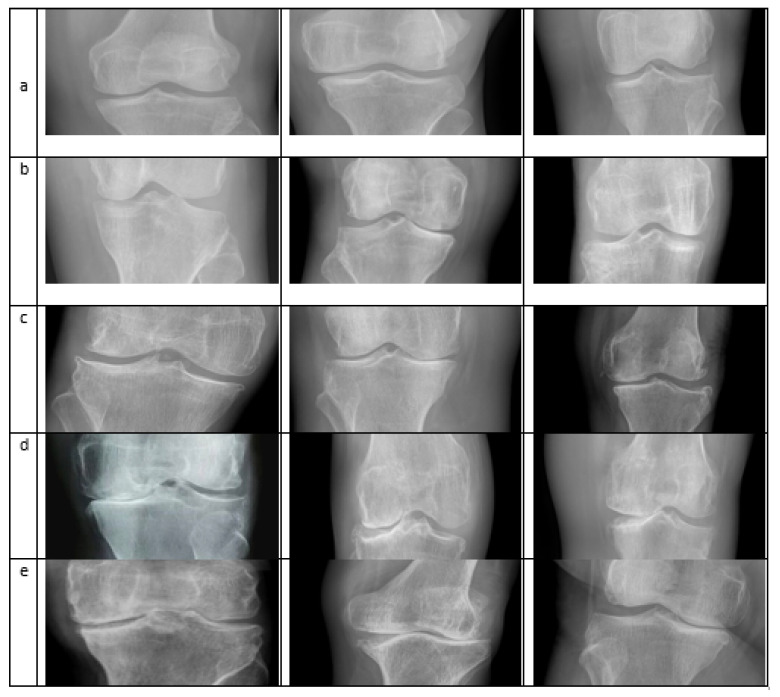
Different images taken from the Dataset: row (**a**) Normal Images; (**b**) Doubtful Images; (**c**) Mild; (**d**) Moderate; (**e**) Severe [48].

The Knee Osteoarthritis Severity Grading dataset, known as the Mendeley dataset IV [47], is used. The experiment was performed on a system Core-i7-7700K 4-core 4.2 GHz with 32 GB RAM (Intel Corporation, Santa Clara, CA, USA) and with NVIDIA Titan V providing 12 GB memory (Nvidia Corporation, Santa Clara, CA, USA). The dataset was collected from different hospitals. X-ray images were taken from the machine, PROTECT PRS 500EX-ray. All the images were in grayscale form and were labeled manually according to KL’s grading system. A total of 500 images were used to train the classifier, of which 100 images showed healthy knees without KOA. For each Grade, according to KL, 100 images were used for the training. A five-fold validation was used, such as (50,50), (25,75), (30,70), (40,60), (20,80); here, an individual set represents the training and testing data respectively for each Grade and the last set is for the healthy class. The algorithm produces an accuracy of 98% for five-fold validation using the KNN algorithm and the combined feature vector of CNN and HOG and SVM with CNN feature vector gives an accuracy of 97.6%. The sample images from different classes are shown in Figure 4.

### 4.2. Results

**Figure 5:**
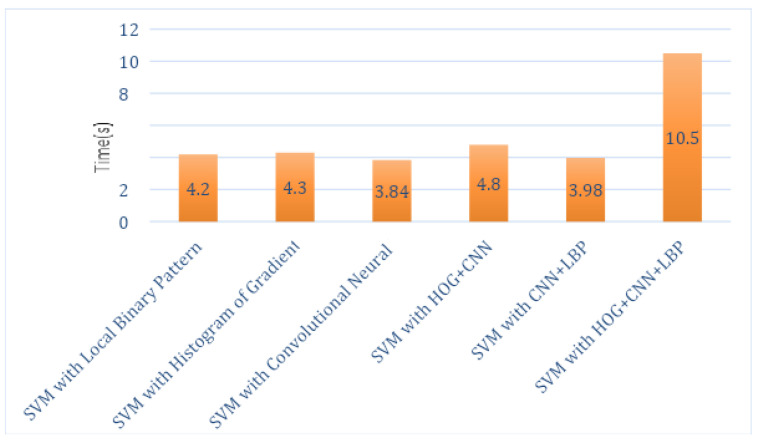
Execution Time Analysis of SVM Classifier.

**Figure 6:**
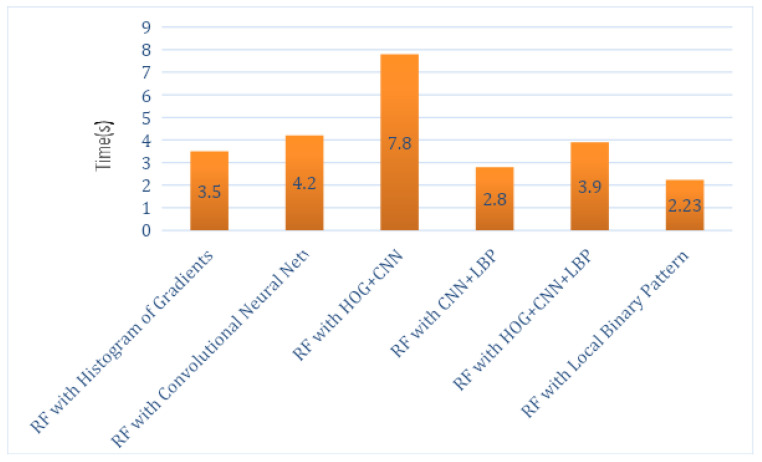
Execution Time Analysis of RF.

**Figure 7:**
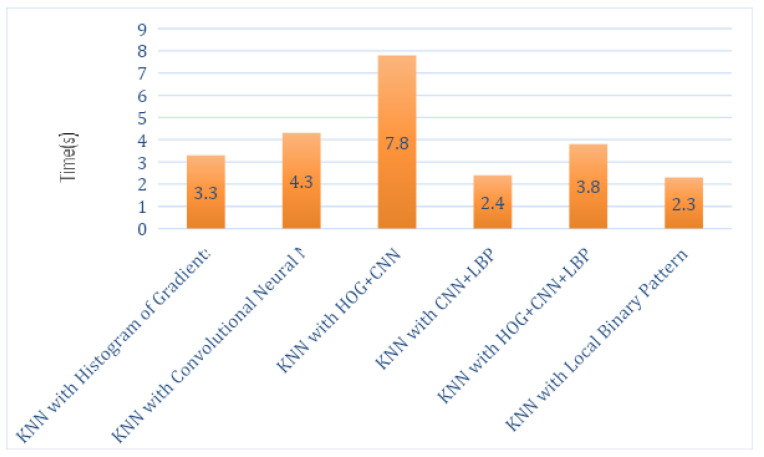
Execution Time Analysis of KNN.

The total execution times of this hybrid proposed system for each feature descriptor with all classifiers, such as SVM, RF and KNN, are computed as shown in Table 2, Table 3 and Table 4. Analysis graphs are shown in Figure 5, Figure 6 and Figure 7. The time taken by the SVM classifier with LBP is 4.2 s, and the shortest time is by SVM_CNN, which is 3.84 s. However, the shortest time of SVM_CNN is also more significant than the shortest time of KNN-LBP, which is 2.3 s.

Hybridized Feature Descriptors: HOG and LBP extracted low-level features while CNN extracted the high-level features such as shear, translate, scale and rotate. The aim is to obtain shape features by combining these hybrid features. These hybridized features, that is, CNN+LBP and CNN+HOG, are used, and then these are classified using SVM, KNN, and RF algorithms. The combined equation of the convolutional neural network and the local binary pattern is given as below:

(9) $$CNN_{LBP}=\sum_{s=1}^{l}\sum_{t=1}^{w}fst.I_{x+s-1,y+t-1}+\;{LBP}_{p,r}$$

(10) $${LBP}_{p,r}=\frac{{\left(I\times fst\right)}_{x,y}-{\left(I\times fst\right)}_{x,y}}{{fst}_{xc-x+1,yc-y+1}},$$

(11) $${LBP}_{p,r}=\sum_{i=0}^{1}k\left(p_{i}-c_{i}\right).2^{i}=I\left(xc,yc\right)\;\&\;k\left(x\right)=\left\{\begin{array}{c} 1,\;\;\;\;\;\;\;\;\;\;\;\;\;if\;x\geq 0\\ 0,\;\;otherwise \end{array}.\right.$$

The combined equation for the convolutional neural network and the histogram of oriented gradients is as below:

(12) $${\left(I_{M,\vartheta }\times K\right)}_{x,y}=\sum_{s=1}^{l}\sum_{t=1}^{w}fst.I_{x+s-1,y+t-1}\;.\;e^{s\vartheta (x+s-1,y+t-1)}.$$

Analysis of Different Combinations of Methods: During the training phase, there were 20 convolutional layers. The size of the kernel for the pooling layer was 2. Convolutional and pooling layers were used alternatively three times. The activation layer was the ReLU layer. At the output layer, the Softmax function was used to extract the features of the knee. The average accuracy attained was 93% within 1870 iterations. The classification algorithms used in the proposed system are SVM, KNN, and RF for five classes, such as healthy and not healthy, which include Grade I, II, III, and IV classes. Firstly, an SVM classifier was trained on the dataset for true positive and true negative classes. We used five-fold cross-validation in which 460 not healthy and 130 healthy images were validated. Table 5, Table 6, Table 7 and Table 8 show the results obtained for the proposed system.

**Figure 8:**
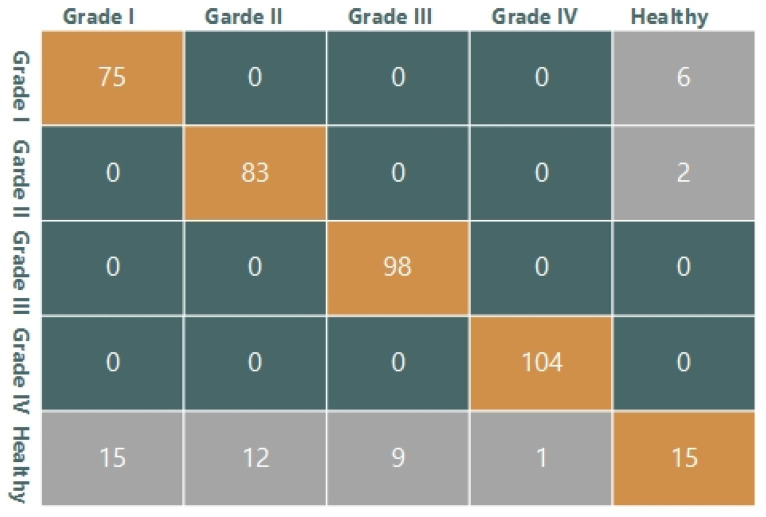
Detailed Confusion Matrix for SVM_LBP.

Other than cross-validation, 420 images were used for testing purposes. Confusion matrices are shown in these tables for all classifiers. The descriptor is shown in Table 5 in the SVM classifier’s confusion matrix with all combinations of features. Therefore, if the total number of images is 420, then SVM_LBP shows 360 True Positive (TP), 15 True Negative (TN), 37 False Positive (FP), and eight False Negative (FN). TP is a number of those images that belong to the positive class and are classified as positive by the system. TN is a number of those images that belong to the negative class, and in actuality, they are also negative. FP is a number of those images that are falsely classified as positive class images. FN is a number of those images that are falsely classified as negative class images. These confusion matrices are based on two classes, that is, healthy and diseased images. Diseased images are further divided into four grades. The detailed confusion matrix for SVM_LBP is shown in Figure 8, where 360 True positive images are divided according to grades of disease. Twenty-seven false-positive images are divided as (15,12,9,1); these images belonged to the healthy knee but they are falsely classified as diseased, that is, Grade I, II, III, and IV. Eight false negative frames were included and six Grade I and two Grade II images were classified as healthy. For all three classifiers used in the proposed system, confusion matrices are given in Table 5, Table 6 and Table 7.

### 4.3. Evaluation Metrics

*Time:* The RF’s shortest time with a local binary pattern that is 2.23 s as described in Table 2, Table 3 and Table 4. The time taken by the SVM classifier with LBP is 4.2 s, and the shortest time by SVM is with CNN, which is 3.84 s. The shortest time by KNN is with LBP, which is 2.3 s.

*Accuracy:* Accuracy is computed to analyze the overall performance of the proposed algorithm on the data. An algorithm based on KNN with a combination of CNN and HOG attained 98% accuracy on five-fold validation and 97% accuracy on cross-validation. The algorithm obtained 91% accuracy for Grade I, 98% accuracy for Grade II, and 99.5% accuracy for Grade III and Grade IV. The equation for accuracy is given below.

(13) $$Accuracy=\frac{TP+TN}{TP+TN+FP+FN}.$$

The true positive, *TP*, refers to the number of images correctly classified as the Non-Healthy class, while false positive, *FP*, refers to the images falsely classified as Non-Healthy. The false negative, *FN*, refers to the frames that our proposed algorithm failed to detect as Non-Healthy but were in fact non-Healthy. *FP* refers to those frames that are falsely classified as non-healthy, but actually were healthy. After the KNN with CNN and HOG, the Random Forest classifier performed well on CNN extracted features by providing an accuracy of 93.09%.

*Recall*: Recall R is calculated and represents the percentage of images that were Non-Healthy that the system recalled. The highest Recall was obtained for the same combination, which was 97.95%. The equation of Recall is given below:

(14) $$R=\frac{TP}{TP+FP}\times 100.$$

**Figure 9:**
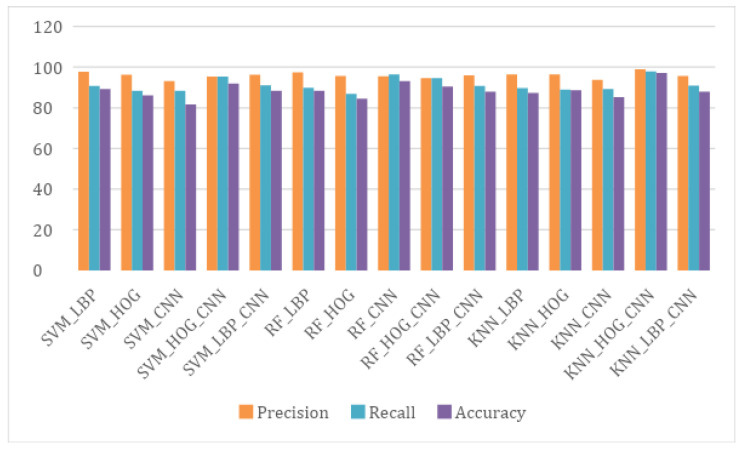
Comparison among the Proposed Algorithms to Select the Best.

*Precision:* Precision P is calculated and represents the percentage of those accurately classified by the proposed system. The highest precision value obtained for the KNN classifier using Convolutional Neural Network with Histogram of Oriented Gradient was 98.96%. A comparison of different proposed algorithms is given in Table 8 in order to select the best and a graphical representation is shown in Figure 9. The average and standard deviations of all the proposed algorithms are also reported in Table 8. The equation of precision is given below:

(15) $$P=\frac{TP}{TP+FN}*100.$$

### 4.4. Comparison with State-of-the-Art

A comparison with the state-of-the-art is performed to analyze the performance of our proposed algorithm. The analysis is shown in Table 9. It is shown that all the existing algorithms are less robust and classify knee disease with less accuracy. The random forest-based technique is proposed in [53] using the images of patients collected from the hospital. It achieved 72.95% accuracy and 76.12% precision. CNN based methods for knee disease detection have been proposed in [15,16,54], using same dataset, that is, OAI and MOST. They attained 61.78%, 66.68%, and 67.49% accuracies. The VGG-19 based technique has been used in [55], achieving an accuracy of 69.70% using the OAI dataset. CNN and LSTM based knee severity disease classification was performed in [56] using the OAI dataset and attaining 75.28% accuracy. The maximum accuracy is attained by [57] based on the LSVM classifier. They attached the sensors to the patient’s knee to collect the data using VAG signals. However, our proposed algorithm has attained 97.14% accuracy and 98.96% precision, which is the best result among the reported existing techniques. The accuracy of different gradings of knee Osteoarthritis (Grade I, II, III, IV) is represented in Table 10.

## 5. Conclusions

A novel knee osteoarthritis detection technique is presented for early-stage prediction. To achieve this goal, deep learning-based feature descriptors are utilized on X-ray images. The image is taken from the Mendeley Dataset VI for training and testing. The proposed model feature is extracted from the region of interest using joint space width by CNN with LBP and CNN with HOG. The multi-class classifiers, that is, SVM, RF, and KNN, are used to classify KOA according to the KL system. Five-Fold Validation and Cross-Validation are performed on the images. The proposed algorithm gives a 97.14% accuracy on cross-validation and a 98% accuracy on five-fold validation. In the future, the proposed model can also be merged with models for the hybrid and diverse detection of different diseases other than of the knee. Further, this can also be exploited using feature fusion methods for the detection and classification of other diseases.

## Figures and Tables

**Table 1:** Summary of recent studies on the detection of Knee Osteoarthritis.

Reference	Dataset	Accuracy	Findings	Contributions
[37]	74 Moderate Knee Osteoarthritis Patients Images	95%	Cross Function and Inverse Dynamics Computed the Knee Moments Outcome efficiently	Only moderate cases were used
[38]	20 KOA and 20 Healthy Knee	95%	Optimized results obtained by Focused Rehabilitation	Patients had only single joint disease
[39]	23 KOA Images and 12 Healthy Images	95%	Results were optimized using IDEEA3 for KOA Anlaysis	Five parameters were considered for measurement of KOA patients to record space
[40]	45 Healthy (18 Males and 25 Females) 100 KOA Patients (45 Males and 55 Females)	98%	Gender is Key Factor in Analysis of KOA	Considered only knee joint kinematics
[41]	91 KOA Patients (22 Males and 29 Females)	97%	KOA patients have greater risk of falling	Selection bias probability
[42]	17 KOA Patients, SRKI and 36 KOA, NSRKI	95%	SRKI cause changes in joints position	Considered KOA patients who were in medical care
[43]	110 KOA Patients (29 youngers, 27 Health Control, 28 Moderate, 26 Severe)	93%	Enhanced KAM was seen in KOA patients	Cross Validation to check the impact of undiagnosed KOA in healthy people
[44]	43 KOA Patients	94%	Gait trail was considered in which only KAM was reappearing	Number of participants was small
[45]	137 KOA Patients	96%	Positive correlation among severe pain and KAM impulse	Study design was cross-sectional

**Table 2:** Execution Time by SVM Classifier Using LBP, CNN and HOG.

Classifier Name	Time in Seconds
SVM with Local Binary Pattern	4.2 s
SVM with Histogram of Oriented Gradients	4.3 s
SVM with Convolutional Neural Network	3.84 s
SVM with HOG + CNN	4.8 s
SVM with CNN + LBP	3.98 s
SVM with HOG + CNN + LBP	10.5 s

**Table 3:** Execution Time by RF Classifier Using LBP, CNN, and HOG.

Classifier Name	Time in Seconds
RF with Histogram of Oriented Gradients	3.5 s
RF with Convolutional Neural Network	4.2 s
RF with HOG + CNN	7.8 s
RF with CNN + LBP	2.8 s
RF with HOG + CNN + LBP	3.9 s
RF with Local Binary Pattern	2.23 s

**Table 4:** Execution Time by KNN Classifier Using LBP, CNN, and HOG.

Classifier Name	Time in Seconds
KNN with Histogram of Oriented Gradients	3.3 s
KNN with Convolutional Neural Network	4.3 s
KNN with HOG + CNN	7.8 s
KNN with CNN + LBP	2.4 s
KNN with HOG + CNN + LBP	3.8 s
KNN with Local Binary Pattern	2.3 s

**Table 5:** Confusion Matrix for SVM with other Feature Descriptors.

	SVM_LBP	SVM_HOG	SVM_CNN	SVM_HOG_CNN	SVM_LBP_CNN
	**P N**	**P N**	**P N**	**P N**	**P N**
P	360 37	342 45	328 43	348 17	356 35
N	8 15	13 20	24 25	17 38	14 15

**Table 6:** Confusion Matrix for RF with other Feature Descriptors.

	RF_LBP	RF_HOG	RF_CNN	RF_HOG_CNN	RF_LBP_CNN
	**P N**	**P N**	**P N**	**P N**	**P N**
P	355 40	332 50	345 13	357 20	353 36
N	9 16	15 23	16 46	20 23	15 16

**Table 7:** Confusion Matrix for KNN with other Feature Descriptors.

	KNN_LBP	KNN_HOG	KNN_CNN	KNN_HOG_CNN	KNN_LBP_CNN
	**P N**	**P N**	**P N**	**P N**	**P N**
P	350 40	345 43	330 40	383 8	351 35
N	13 17	13 19	22 28	4 25	16 18

**Table 8:** Comparison of Different Proposed Algorithms.

Method	Average	Standard Deviation	Precision	Recall	Accuracy
SVM_LBP	0.5342	0.0581	97.82%	90.68%	89.28%
SVM_HOG	0.4993	0.0631	96.33%	88.37%	86.19%
SVM_CNN	0.5521	0.0913	93.18%	88.40%	81.66%
SVM_HOG_CNN	0.5330	0.0632	95.34%	95.34%	91.90%
SVM_LBP_CNN	0.4783	0.0634	96.21%	91.04%	88.33%
RF_LBP	0.5432	0.0599	97.52%	89.87%	88.33%
RF_HOG	0.4231	0.0653	95.67%	86.91%	84.52%
RF_CNN	0.4323	0.0685	95.56%	96.36%	93.09%
RF_HOG_CNN	0.4345	0.0654	94.69%	94.69%	90.47%
RF_LBP_CNN	0.4594	0.0601	95.92%	90.74%	87.85%
KNN_LBP	0.4534	0.0610	96.41%	89.74%	87.38%
KNN_HOG	0.4958	0.0611	96.36%	88.91%	88.66%
KNN_CNN	0.4789	0.0672	93.75%	89.18%	85.23%
KNN_HOG_CNN	0.5412	0.0580	98.96%	97.95%	97.14%
KNN_LBP_CNN	0.4345	0.0632	95.64%	90.93%	87.85%

**Table 9:** Comparison of Different Proposed Algorithms.

Method	Recall	Precision	Accuracy	Data Set
CNN [16]	62	58	61.78	OAI and MOST
DeepCNN [58]	-	-	66.68	OAI and MOST
Siamese CNNs [15]	-	-	67.49	OAI and MOST
VGG-19 [55]	-	-	69.70	OAI
CNN-LSTM [56]	-	-	75.28	OAI
Faster R-CNN [54]	-	-	74.30	-
LASVM [57]	-	-	86.67	VAG Signals
RF [53]	60.49	67.12	72.95	Hospital Images
The Proposed Algorithm	97.95	98.96	97.14	Mendeley Dataset IV

**Table 10:** Accuracy on different gradings of knee Osteoarthritis (Grade I, II, III, IV).

Grade	Accuracy (%)
I	91
II	98
III	99.5
IV	99.5

## Data Availability

Publicly available datasets were analysed in this study. This data can be found here: https://www.frontiersin.org/articles/10.3389/frobt.2020.591827/full (accessed on 23 June 2020) and https://data.mendeley.com/datasets/t9ndx37v5h/1#folder-18a3659a-1fa2-4340-b7bb-526fb81006f6 (accessed on 23 June 2020).
